# Repurposing and Revival of the Drugs: A New Approach to Combat the Drug Resistant Tuberculosis

**DOI:** 10.3389/fmicb.2017.02452

**Published:** 2017-12-11

**Authors:** Divakar Sharma, Yogesh K. Dhuriya, Nirmala Deo, Deepa Bisht

**Affiliations:** ^1^Department of Biochemistry, National JALMA Institute for Leprosy and Other Mycobacterial Diseases, Agra, India; ^2^Interdisciplinary Biotechnology Unit, Aligarh Muslim University, Aligarh, India; ^3^Developmental Toxicology Laboratory, Systems Toxicology and Health Risk Assessment Group, CSIR-Indian Institute of Toxicology Research (CSIR-IITR), Lucknow, India

**Keywords:** drug resistance tuberculosis, repurposing, revival of drugs, synergistic effect, proteomics and bioinformatics

## Abstract

Emergence of drug resistant tuberculosis like multi drug resistant tuberculosis (MDR-TB), extensively drug-resistant tuberculosis (XDR-TB) and totally drug resistant tuberculosis (TDR-TB) has created a new challenge to fight against these bad bugs of *Mycobacterium tuberculosis*. Repurposing and revival of the drugs are the new trends/options to combat these worsen situations of tuberculosis in the antibiotics resistance era or in the situation of global emergency. Bactericidal and synergistic effect of repurposed/revived drugs along with the latest drugs bedaquiline and delamanid used in the treatment of MDR-TB, XDR-TB, and TDR-TB might be the choice for future promising combinatorial chemotherapy against these bad bugs.

## Introduction

### Current Scenario

*Mycobacterium tuberculosis* is a deadly infectious pathogen causing tuberculosis (TB) worldwide. According to WHO (2016) 10.4 million new cases with 1.5 million deaths including 0.4 million individuals with HIV-TB co-infection were reported globally (WHO, 2016). Although TB can be cured by chemotherapy, but the emergence of drug resistant tuberculosis [such as multidrug-resistant tuberculosis (MDR-TB), extensively drug-resistant tuberculosis (XDR-TB) and totally drug resistant tuberculosis (TDR-TB)] has created a new challenge to combat the adverse situation of the disease. Since the decade rates of antibiotic resistance to first and second line anti-TB drugs are dramatically increasing (Cambau et al., 2015). Due to various complexities and high burden of HIV-TB co-infection treatments of MDR-TB, XDR-TB, and TDR-TB are problematic. Apart from the genomics studies various proteomics as well as bioinformatics studies regarding to the drug resistance tuberculosis were accumulated in the last decade (Jiang et al., 2007; Zhang and Yew, 2009; Sharma et al., 2010, 2014, 2015a,b, 2016a,b, 2017; Kumar et al., 2013; Lata et al., 2015a,b; Singh et al., 2015; Sharma and Bisht, 2016, 2017a,b,c;) and suggested the novel drug resistance mechanisms/markers/targets for potential therapeutics in near future. Proteomics and bioinformatics play a crucial role in the diagnostics and therapeutics against the emerging bad bug of tuberculosis. Apart from the expression proteomics studies cited above we have reported that Rv0148 (Putative short-chain type dehydrogenase/reductase) and Rv3841 (ferritin protein) have potentially involved in aminoglycosides resistance (Sharma et al., 2015b, 2016a) and could be the potential diagnostics and therapeutics against the second line of drug resistance. To combat these worsened situations (MDR-TB, XDR-TB, and TDR-TB) new and effective drugs and diagnostics are urgently needed. However, as we know that development of new drugs and diagnostics are enormously expensive and time consuming process.

### Repurposing and Revival of Drugs: A Chemotherapeutic Option against Drug Resistant Tuberculosis

Repurposing and revival of the drugs are the new trends/options or one of the pharmaceutical strategies to treat the particular disease that are already FDA approved for other diseases (Tsukamura, 1980; Van Deun et al., 2010) and also for tuberculosis earlier, in this antibiotics resistance era or in the situation of global emergency. Many compounds in TB advanced clinical trials are the molecules that were formerly used to treat other infectious diseases/tuberculosis earlier and now they have been repurposed for treatment of TB (Nayer and Steinbach, 1939; Tsukamura, 1980; Van Deun et al., 2010; Hasse et al., 2014).

Sulfonamides and sulphanilamide were first used in 1930s as an anti-TB drug (Nayer and Steinbach, 1939) but its use was discontinued due to its lesser efficacy as compared to first line drugs (streptomycin and isoniazid). Revival of sulfamethoxazole (SMX) in TB was first pointed out by its efficacy to prevent the TB infection in HIV patients whose receiving trimethoprim/sulfamethoxazole (TMP/SMX) to prevent other infection such as *Pneumocystis jirovecii* (Hasse et al., 2014). In a Nigerian trial study on patients of HIV-MDR-TB coinfetion, efficiency of MDR-TB treatment by TMP/SMX confirmed a significantly shorter time to sputum conversion in these patients (Oladimeji et al., 2014). Sulfadiazine is an anti-leprosy drug which is repurposed in the treatment of MDR-TB and XDR-TB. Brouqui et al. (2013) suggested that sulfadiazine regimen is safe and effective against MDR-TB and TDR-TB treatment (Ameen and Drancourt, 2013; Brouqui et al., 2013).

Clofazimine (CZM) is one of the repurposed molecules, has been initially used as an anti-leprosy drug since half the century. It was recently repurposed for managing the treatment of MDR-TB (Van Deun et al., 2010). CZM is recommended as a second-line anti-TB drug and used in combination with other anti-TB drugs for the treatment of drug-resistant tuberculosis. CZM-containing regimen can cure MDR-TB cases in 9–12 months. In *M. tuberculosis*, CZM appears to act as a prodrug, which is reduced by type 2-NADH dehydrogenase, to release reactive oxygen species (ROS) upon reoxidation by oxygen (O_2_) (Yano et al., 2011). CZM, exhibits noticeable anti-mycobacterial and anti-inflammatory activity by inhibition of phospholipase and effects on potassium transporters, respectively (Steele et al., 1999; Cholo et al., 2006). Previous published studies have reported that CZM has good quality efficacy and little toxicity against drug-resistant mycobacterial strains in animal models, which suggested, CZM as a promising anti-TB drug for the management of MDR-TB (Van Deun et al., 2010; Cholo et al., 2012). Recently, numerous observational studies have reported that CZM including regimens provided a useful role in the treatment of patients with MDR-TB (Cholo et al., 2006; Van Deun et al., 2010).

Linezolid, an oxazolidinone antibiotic which is used for treatment of gram-positive bacterial infections (Till et al., 2002; Yanagihara et al., 2002), has now potentially repurposed for the treatment of drug resistant TB (MDR-TB and XDR-TB) (Fortún et al., 2005). Linezolid is an effective anti-TB drug for treating MDR-TB and XDR-TB with various side effects such as neurotoxicity and hematologic toxicity (Tang et al., 2015). Schecter et al. (2010) suggested that linezolid had low rates of discontinuation, well tolerated and good efficacy in the treatment of MDR-TB. Most recently in a case study Jaspard et al. (2017) reported, bedaquiline and linezolid drug combination might be safe for XDR-TB in the late third trimester of pregnancy or pregnant woman. Pregnant woman gave birth to a child without abnormalities follow-up of the fetal showed that no fetal toxicities upto 2 years after the delivery (Jaspard et al., 2017).

Minocycline is also one of the repurposed molecules, has been initially used in the treatment of leprosy since the 1980s (Tsukamura, 1980). In 2008 it was repurposed for managing the treatment of XDR-TB patient in Japan (Kawada et al., 2008). Combinatorial therapy of amoxicillin/clavulanic acid along with other second-line drugs has been used in the treatment of MDR-TB. It’s cheaper cost and less toxicity has made the drug of choice in WHO group five drugs (Cassir et al., 2014). Recently, combinatorial treatment by amoxicillin/clavulanic acid and carbapenems has reduced the *M. tuberculosis* load (Diacon et al., 2016). Hugonnet et al. (2009) reported the *in vitro* activity of meropenem combined with clavulanate against XDR strains and paying attention to repurpose these beta-lactams as new anti-TB drugs (Hugonnet et al., 2009). However, carbapenems have been used successfully as part of salvage therapies for XDR patients, they have to be administered intravenously (Tiberi et al., 2016). Recently, an early bactericidal activity-Phase II (EBA Phase II) clinical trial has validated the promising potential of a carbapenem combined with amoxicillin and clavulanic acid for TB treatment (Diacon et al., 2016).

Singhal et al. (2014) reported that the FDA-approved antidiabetic drug metformin (MET) inhibits the intracellular mycobacterial growth by inducing mitochondrial ROS production, restricts disease immunopathology, enhances the efficacy of other anti-TB drugs and could be used as combinatorial therapy against the drug resistant tuberculosis. Gupta et al. (2013) suggested that tuberculosis treatment shortening by verapamil as an adjunctive therapy in mice has opened the direction for future research on verapamil and other efflux pump inhibitors in human tuberculosis. Gupta et al. (2014) also reported that efflux inhibition with verapamil tremendously decreases the MIC of bedaquiline and CZM to *M. tuberculosis* and suggested the synergistic effects of verapamil and bedaquiline in an animal model of TB infection. Recently Dutta et al. (2016) reported that statin a lipid-lowering drug (repurposed for TB treatment) when added to the first-line antitubercular drugs, reduces the lung bacillary burden in chronically infected mice.

Host-directed therapies are important adjunctive therapies for tuberculosis treatment that expanded by host immune effector mechanisms. Recently Gupta et al. (2017) showed that denileukin diftitox potentiates standard TB treatment in the mouse model, which might be due to depletion of T-regulatory and myeloid-derived suppressor cells during TB infection.

### Synergistic Effects of Repurposed/Revived Drugs: With Others Anti-TB Drugs Used in Treatments of Tuberculosis Bad Bug

Synergistic effect of repurposed/revived drugs with other anti-TB drugs has been used for the future selection of these drugs in the WHO regimen which could be used in the treatment of MDR-TB, XDR-TB, and TDR-TB. In a study Zhang et al. (2015) suggested that CZM in combination with ethambutol (EMB) and moxifloxacin (MOX) may be a potential drug regimen for the treatment of MDR-TB. Synergistic effect of SMX has been reported *in vitro* with rifampicin (Macingwana et al., 2012). Tasneen et al. (2011) suggested CZM was the best third drug in combination with bedaquiline and pyrazinamide for the treatment of MDR-TB and a good example of drug synergism. Partial synergistic effect was oveserved between linezolid and capreomycin and suggested the efficacy of this combinatorial therapy against *M. tuberculosis* (Zhao et al., 2016). Most recently synergistic effect of bedaquiline and linezolid combinatorial therapy has been reported in XDR-TB of pregnant woman which is a good symbol of synergy for the last line of drugs (Jaspard et al., 2017). In a study synergistic effect carbapenems with rifampicin have been reported against *M. tuberculosis* (Kaushik et al., 2015).

## Conclusion and Future Perspective

On the basis of the reported literature and studies (regarding to proteomics, bioinformatics, and repurposing/revival of drugs) cited above we suggest that proteomics and bioinformatics play a crucial role in the exploration of diagnostics, therapeutics and mechanism of resistance against drug resistance tuberculosis. Repurposing and revival of the drugs is an alternative strategy to combat from this inadequate situation of drug resistance tuberculosis in this antibiotic resistance era. Synergistic effect of repurposed/revived drugs (sulfamethoxazole, sulfadiazine, clofazimine, linezolid, minocycline, amoxicillin/clavulanic acid, and carbapenems like meropenem) along with the latest drugs (bedaquiline and delamanid) used in the treatment of MDR-TB and XDR-TB might be one of the promising combinatorial chemotherapy for the treatment of MDR-TB, XDR-TB, and TDR-TB. Synergistic effect of these repurposed/revived drugs with bedaquiline and delamanid combinatorial therapy could be added a different perspective over the existed literature.

## Author Contributions

DS design the concept and wrote the manuscript. DS, YD, ND, and DB finalized the manuscript.

## Conflict of Interest Statement

The authors declare that the research was conducted in the absence of any commercial or financial relationships that could be construed as a potential conflict of interest.
